# Comprehensive analysis of the SLC16A gene family in pancreatic cancer via integrated bioinformatics

**DOI:** 10.1038/s41598-020-64356-y

**Published:** 2020-04-30

**Authors:** Shan Yu, Yanshuang Wu, Chunlong Li, Zhaowei Qu, Ge Lou, Xiaorong Guo, Jingjing Ji, Nan Li, Mian Guo, Maomao Zhang, Lei Lei, Sheng Tai

**Affiliations:** 1 Department of Pathology, the Second Affiliated Hospital of Harbin Medical University, Harbin, 150001 China; 2 Department of Histology and Embryology, Harbin Medical University, Harbin, 150001 China; 3 Department of Hepatobiliary and Pancreatic Surgery, the Second Affiliated Hospital of Harbin Medical University, Harbin, 150001 China; 4 Department of Hepatobiliary and Pancreatic Surgery, Harbin Medical University Cancer Hospital, Harbin, 150001 China; 5 Department of Pathology, the Fourth Affiliated Hospital of Harbin Medical University, Harbin, 150001 China; 6 Department of Neurosurgery, the Second Affiliated Hospital of Harbin Medical University, Harbin, 150001 China; 7 The Key Laboratory of Myocardial Ischemia, Department of Cardiology, the Second Affiliated Hospital of Harbin Medical University, Harbin, 150001 China; 8 The Key Laboratory of Preservation of Human Genetic Resources and Disease Control in China, Harbin Medical University, Ministry of Education, Harbin, 150001 China

**Keywords:** Tumour biomarkers, Microarrays

## Abstract

SLC16A family members play crucial roles in tumorigenesis and tumor progression. However, the exact role of distinct members in the SLC16A family in human pancreatic cancer remains unclear. Integrated bioinformatics analysis for the identification of therapeutic targets for certain cancers based on transcriptomics, proteomics and high-throughput sequencing could help us obtain novel information and understand potential underlying molecular mechanisms. In the present study, we investigated SLC16A family members in pancreatic cancer through accumulated data from GEO (Gene Expression Omnibus), TCGA (The Cancer Genome Atlas) and other available databases. The expression profile, clinical application significance and prognostic value of the SLC16A family for patients with pancreatic cancer were explored. SLC16A1, SLC16A3 and SLC16A13 exhibited biomarker potential for prognosis, and we further identified their related genes and regulatory networks, revealing core molecular pathways that require further investigation for pancreatic cancer.

## Introduction

The solute carrier (SLC) group of membrane transport proteins includes over 400 members organized into 65 families^1^. Most members of the SLC group are located in the cell membrane^2^. The SLC16A family of monocarboxylate transporters (MCTs) is a subfamily of solute carriers that transport monocarboxylate molecules, including L-lactate and pyruvate, across cell membranes^3^. They are known to play an important role in cell metabolism, such as aerobic glycolysis and pH homeostasis^4^. Currently, 14 gene members named SLC16A1 to SLC16A14 have been identified to have the presence of two highly conserved sequence regions located in TM1 and TM5, respectively, by topology study^5^. However, only four proteins are confirmed and encoded by the SLC16A gene family. The four coded proteins were named MCT1 (SLC16A1), MCT4 (SLC16A3), MCT2 (SLC16A7) and MCT3 (SLC16A8)^4^. The functions of the rest of the members of the SLC16A gene family have not been fully revealed by experiments in human diseases^6^.

Current research progress on the MCT family is mainly based on the findings of MCT1-4. MCT1-4 exhibit broad substrate specificity and are also involved in the transport of other monocarboxylic metabolites, e.g., pyruvate, ketone bodies and specific drugs^7^. MCT1 is ubiquitously expressed in almost all human cells, while MCT2 is mainly found in neurons, liver and kidney and facilitates L-lactate entry into or efflux out of cells depending on the metabolic requirement. MCT3 is only detected in retinal histology, and MCT4 is highly expressed in glycolytic and anaerobic tissues, such as skeletal muscle and mammalian glands, both with the function of L-lactate export^8^. Regarding the affinity of L-lactate, MCT4 has a lower Km (20~35 mM) than MCT2 (~0.7 mM), MCT1 (3~5 mM) and MCT3 (~6 mM)^4,8,9^. These MCTs catalyze the diffusion of lactate without any energy due to the accumulation or exclusion of the lactate anion through a pH gradient^10,11^. Moreover, the glycolytic phenotype is also supported by MCTs. In cancer cells, acidification by lactic acid production is believed to be an essential metabolic process for tumor cell growth, migration and angiogenesis^12^.

As monocarboxylate transporters mainly participate in the metabolism of glucose, which is heavily relied on by malignant tumors, a deep understanding of this membrane protein family has become critical for cancer metabolism-related research^13^. Current studies have demonstrated that the aberrant expression of SLC16A gene family members occurs in various types of malignant tumors, regulating cell migration, invasion and proliferation^14–16^.

Pancreatic cancer is one of the most fatal human malignant diseases with poor clinical outcomes^17^. The five-year survival rate is not improved compared with other cancers due to the lack of clinical symptoms at an early stage and effective gene or chemotherapy once in the late stage^18^. As pancreatic cancer is a kind of highly glycolytic malignant tumor that maintains lactic acid effluxes to maintain intracellular pH homeostasis through MCTs, inhibiting lactic acid efflux is a promising therapeutic target against cancers^19,20^.

Currently, genomic network analysis from large databases, which enable us to understand certain gene family functions, has been widely accepted^21^. Although some independent experiments illustrate the potential of MCT as a target in cancer research, our knowledge of the SLC16A family in human malignancies is quite largely unexplored.

In our present study, we are trying to perform a comprehensive analysis to investigate the best biological understanding of the SLC16A family members according to our up-to-date knowledge. Through integrated bioinformatics, we hope that our research will provide novel molecular therapeutic targets and their potential biological functions for pancreatic cancer patients.

## Methods

### SLC16A family member expression profile by pan-cancer analysis

To analyze the expression of the SLC16A family gene members in various cancers, data from Oncomine, TCGA, and the Genotype-Tissue Expression (GTEx) databases were obtained for expression profile visualization. Oncomine gene expression microarray datasets (www.oncomine.org) are a well-known web-based genome-wide expression platform^22^, which is applied to compare the transcriptome expression profile data of each SLC16A family member in cancers vs normal tissues. “SLC16A” was set as a key search word, and “cancer vs normal tissues analysis” was set as a primary filter. The cancer subtypes with P-values and expression fold changes compared vs normal tissues were extracted. P < 0.05, 1.5-fold change and expressed gene rank in the top 10% were input as our selection threshold. All studies that met our request were extracted with the target gene expression in both control tissue and pancreatic cancer tissue. The bar chart of these studies was plotted by the log2 median-centered intensity and case number. Moreover, we extracted SLC16A family RNA-seq data in both pancreatic cancer and normal tissue from TCGA and GTEx by Gene Expression Profiling Interactive Analysis (GEPIA)^23^. A heatmap was plotted to demonstrate the RNA-seq data using log2(TPM + 1).

#### Kaplan-Meier overall survival analysis

To analyze the independent prognostic value of SLC16A family members in pancreatic adenocarcinoma, the Kaplan-Meier plot was applied from the living survival data extracted from TCGA-PAAD by GEPIA to display overall survival (OS) separated by median RNA-seq expression value transcripts per million (TPM). The total number of patients with PAAD was 178, and a 95% confidential interval was also demonstrated. A log-rank P-value <0.05 was considered statistically significant. As the GEO database contains microarray analysis results of a cohort of patients with pancreatic cancer, we chose GSE28735, which is a well-known pancreatic cancer research study including the gene expression profile of 45 pairs of pancreatic tumor and adjacent nontumor tissues using Affymetrix GeneChip Human Gene 1.0 ST arrays^24^. The overall-survival KM plot of the GEO dataset was obtained and analyzed by shinyGEO, which is a web-based application for analyzing gene expression with clinical outcomes^25^. The microarray probe for SLC16A1 is 7918622 and 8010770 for SLC16A3.

#### Immunohistochemistry (IHC) from the HPA

The Human Protein Atlas (HPA) is an open systematic accessible study on the human proteome using antibody proteomics in cell and tissue samples (https://www.proteinatlas.org/)^26^. Most of the protein-coding genes in different cancers and normal tissues could be investigated by this user-friendly online interactive database. In our study, a representative IHC expression pattern for clinical potential application was extracted from the HPA in both normal pancreas tissue and pancreatic adenocarcinoma. A clear contrast between normal and cancer samples with different staining signals was selected for pancreatic cancer prognosis prediction in potential clinical applications. The IHC result for the protein expression pattern was recalculated by two independent pathologists and given remarks as “weak, moderate and strong” for SLC16A1 and SLC16A3. The summary of the staining intensity was plotted in a bar chart.

#### GEO data extraction and identification of DEGs

The GEO database, which contains thousands of public microarrays and sequencing profiles, was searched, and studies with the SLC16A1 and SLC16A3 genes were downloaded (http://www.ncbi.nlm.nih.gov/geo/)^27^. As SLC16A13 was only predicted to encode a protein with relatively low expression in RNA-Seq and no positive research according to our Oncomine analysis, we did not include a GEO study on SLC16A13.

The GSE76675 dataset was designed to investigate the MCT1 (SLC16A1) level in glycolytic and malignant cancer cells through a stable knockdown mediated by the short hairpin RNA method and MCT1 inhibitor (AZD3965) by Affymetrix Human Genome U133 Plus 2.0 Array (GPL570 platform)^28^. However, we only used the data from the scramble shRNA and sh-MCT1 groups (GSM2035675; GSM2035676; GSM2035677; GSM2035678; GSM2035679; GSM2035680).

The GSE63231 dataset was designed to explore the gene expression alterations determined with control or MCT4 (SLC16A3) knockdown in pancreatic cancer cells by specific RNAi in an Illumina HumanHT-12 V4.0 expression beadchip (GPL10558 platform)^29^. Datasets of all three pancreatic cancer cell lines (MiaPaca2, Capan-2, and PL45) were extracted (from GSM1544097 to GSM1544108). The microarray data from the indicated GEO datasets were obtained and further normalized. The R software preloaded with the limma package was applied to calculate the differentially expressed genes (DEGs)^30^. A volcano plot was used to illustrate the DEGs in GSE76675 and GSE63231. Moreover, the top 20 upregulated and downregulated DEGs were plotted by a heatmap generated by the Heml heatmap illustrator^31^.

All genes of the SLC16A family (SLC16A1 to SLC16A14) were also plotted into a heatmap to demonstrate the silencing effect of SLC16A1 or SLC16A3 on other genes in the SLC16A family using the RNA-seq data from the two GEO datasets.

#### Integrated bioinformatics analysis

Gene Ontology (GO) terms (biological process) for all DEGs from GEO datasets were obtained using the GO database version 1.2 (released 2019-02-02) through the Gene Ontology resource (http://geneontology.org)^32^. The Gene Ontology Knowledgebase contains information on the functions of genes of interest, which is widely used in biomedical research.

The enrichment ratio P-value for each GO term was calculated, and all enriched pathways were plotted by using Word cloud for the Enrichment Analysis, version 1.0^33^. FDR < 0.05 was set as a cutoff for significance. KEGG pathway analysis was applied to all DEGs. The online-web tool Metascape (http://metascape.org), which is known as a web portal for gene annotation and a resource for meta-analysis tool, was used to understand common and unique pathways within genes of interest. Metascape was used to obtain the KEGG enrichment results, which were further displayed^34^.

The predicted PPI (protein-protein interaction) was generated by the Search Tool for the Retrieval of Interacting Genes (STRING) web database (version 10.5; http://string-db.org/)^35^. To evaluate the interactive associations within all DEGs in both GEO datasets, the highest confident interaction with an indicated score was selected and clearly mapped. All disconnect dots were excluded. The DEG PPI network was constructed and visualized using Cytoscape software (version 3.5.1; www.cytoscape.org)^36^. The PPI biological process GO terms were also enriched.

## Results

### Differential expression studies of the SLC16A family transcript in pan-cancer

**Figure 1:**
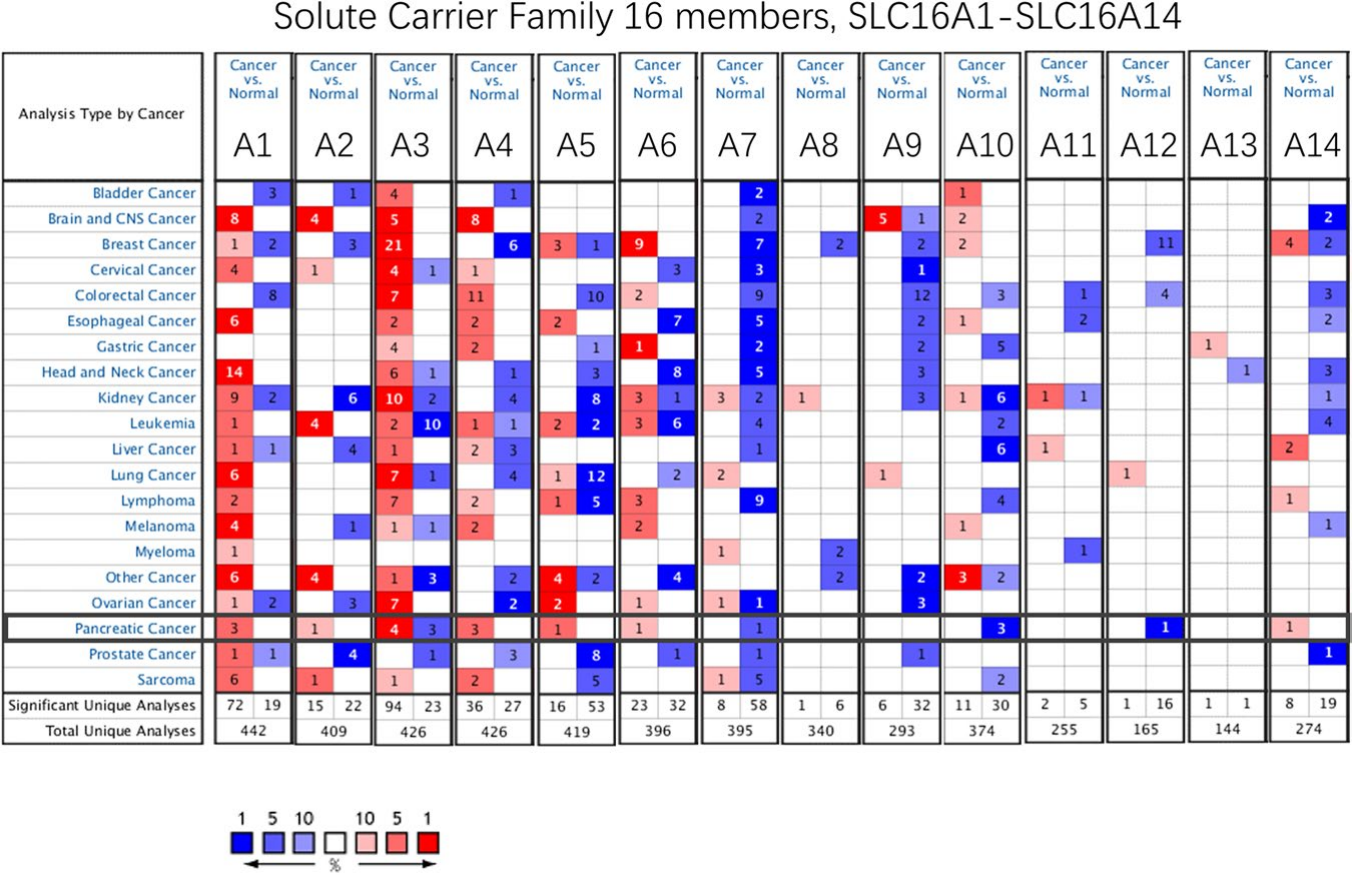
Pan-cancer analysis of SLC16A family genes according to the Oncomine database. Red background with numbers indicates the studies including SLC16A member expression levels meeting our selection standards (with P-values <0.05 and expression fold changes >1.5-fold change and expressed gene rank in the top 10% as our selection threshold) in cancer tissue; Blue (the same selection threshold) in normal tissues. The number for the Significant Unique Analyses indicates that the queried gene is a significantly different gene in studies. The number for the Total Unique Analyses indicates the total number of studies that contain the queried gene.

All fourteen SLC16A family members from SLC16A1 to SLC16A14 were investigated in 20 types of malignancies by the Oncomine database. The Oncomine database contains a total of 442, 409, 426, 426, 419, 396, 395, 340, 293, 374, 255, 165, 144 and 274 different studies involving the genes from SLC16A1 to SLC16A14, respectively (Fig. 1). The significant unique analyses between cancer and normal tissues meeting our selection criteria in Pan-cancer were shown in Fig. 1 bottom cells. The case number in left cell indicates gene up-regulated and in right cell shows the down-regulated case. The counting result was SLC16A1 (72:19), SLC16A2 (15:22), SLC16A3 (94:23), SLC16A4 (36:27), SLC16A5 (16:53), SLC16A6 (23:32), SLC16A7 (8:58), SLC16A8 (1:6) SLC16A9 (6:32), SLC16A10 (11:30), SLC16A11 (2:5), SLC16A12 (1:16), SLC16A13 (1:1), SLC16A14 (8:19).

In detail, in pancreatic cancer, the SLC16A1 and SLC16A4 mRNA expression levels increased in all 3 cases. The expression level of SLC16A10 was down-regulated in 3 independent pancreatic cancer researches.. However, other genes in the SLC16A family met our selection standard, except SLC16A3 (4:3). SLC16A1 has a significantly increased expression level in brain and CNS cancer, esophageal cancer, head and neck cancer, kidney cancer, lung cancer, melanoma, and even sarcoma, indicating its importance in pan-cancer. SLC16A3, more than SLC16A1, shows a significant increase in female reproductive cancer (breast 21:0, cervical 4:1 and ovarian cancer 7:0). In contrast, SLC16A5, SLC16A6, SLC16A7 and SLC16A9 were mainly expressed at lower levels in tumors than in normal tissues.

### SLC16A family expression in pancreatic cancer

**Figure 2:**
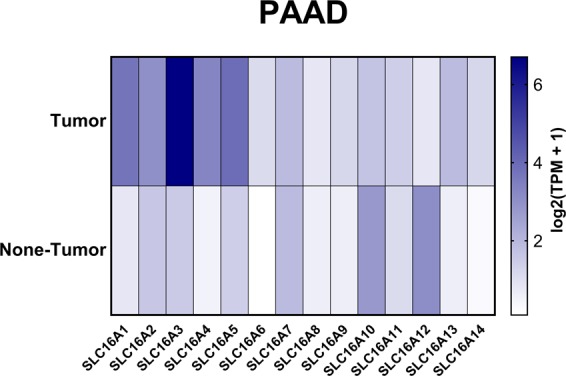
The heatmap of the RNA-Seq profile indicates the expression of SLC16A family members after normalization by log2(TPM + 1) for log-scale compared with pancreatic adenocarcinoma and normal pancreas tissues from TCGA. The cancer abbreviation name is shown according to the TCGA study abbreviation (PAAD = pancreatic adenocarcinoma). The dark color indicates more reads obtained for the gene.

**Figure 3:**
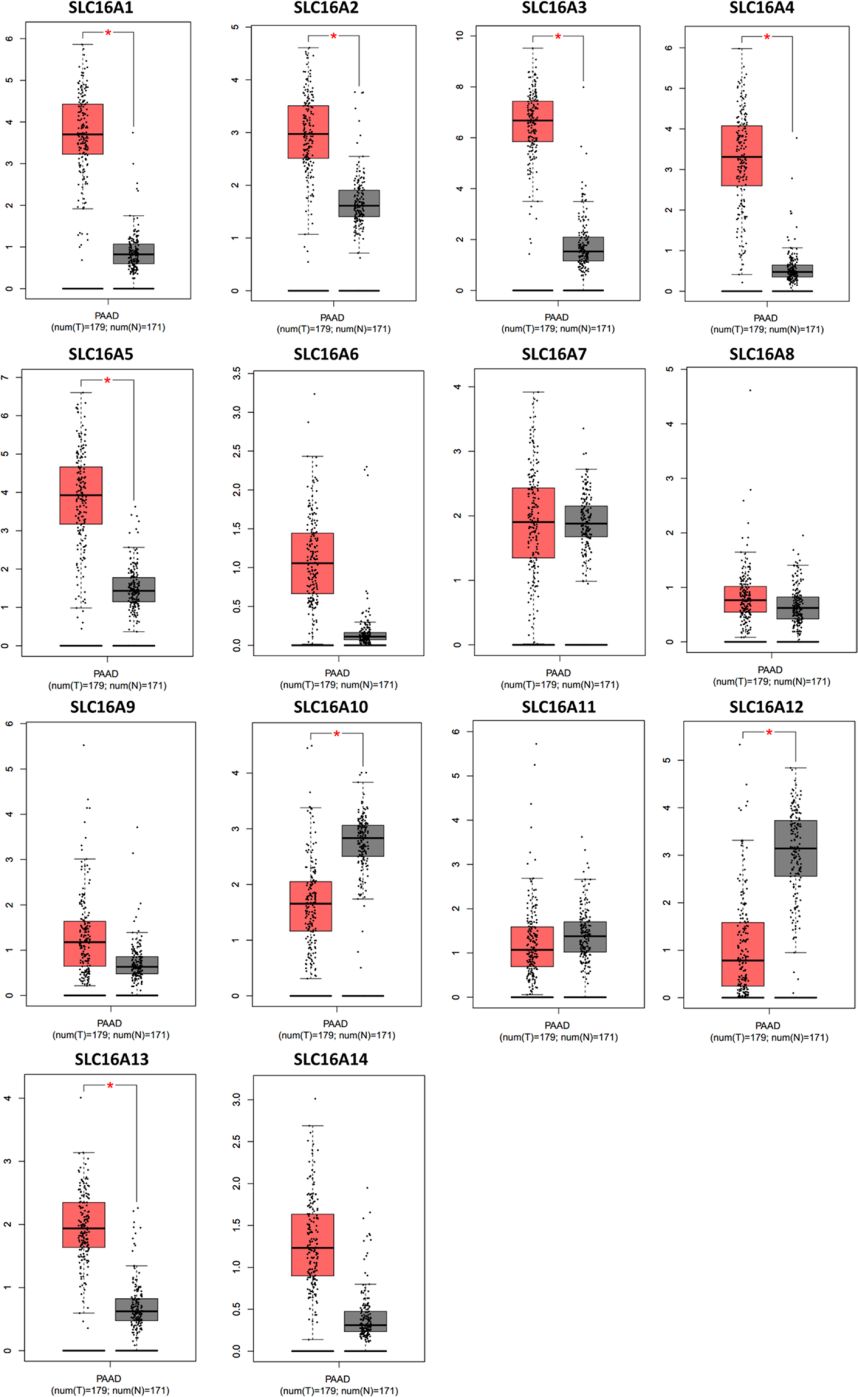
Boxplot of the normalized SLC16A family member expression levels in the distribution profile of patients with PAAD vs the normal pancreas tissue expression distribution profile. PAAD sample number = 179 is in red, normal pancreas tissue number = 171 is in gray. Tumor samples were defined as pancreatic cancer samples, and normal tissue was defined as normal pancreatic tissue from nonpancreatic disease patients. *Statistically significant difference in expression.

The RNA-Seq expression heatmap (Fig. 2) shows the relative mRNA expression within the SLC16A family. We found that SLC16A3 has the highest expression level among the genes in the family, followed by SLC16A1 and SLC16A5. From SLC16A1 to SLC16A5, compared with the normal tissues, the RNA-seq data in PAAD revealed a significant increase. The studies from the Oncomine database also clearly show the differences in increasing levels of SLC16A1 and SLC16A3 between pancreatic cancer and normal tissue (Figure S2). The boxplot of the RNA-Seq expression in 179 pancreatic cancer vs 171 normal pancreas samples demonstrated that SLC16A1, SLC16A2, SLC16A3, SLC16A4, SLC16A5 and SLC16A13 were increased with statistical meaning (Fig. 3). Moreover, SLC16A10 and SLC16A12 were expressed at lower levels in pancreatic cancer. For other SLC16A family members, there is no statistical significance between PAAD and normal tissues. These findings strongly suggest that the SLC16A family may have a huge impact on pancreatic cancers. Moreover, the knockdown of SLC16A1 shows a negative correlation change in SLC16A6 and positive upregulation of SLC16A2 and SLC16A14. The knockdown of SLC16A3 demonstrated a negative change in SLC16A2 (Capan and M2A cell lines), SLC16A5, SLC16A10 and SLC16A14 (PL45 and M2A cell lines) and caused positive changes in SLC16A9 (Capan, M2A and PL45 cell lines). Those gene alternations are demonstrated in heatmaps (Figure S3).

### Prognostic value of the SLC16A family by kaplan-meier analysis

**Figure 4:**
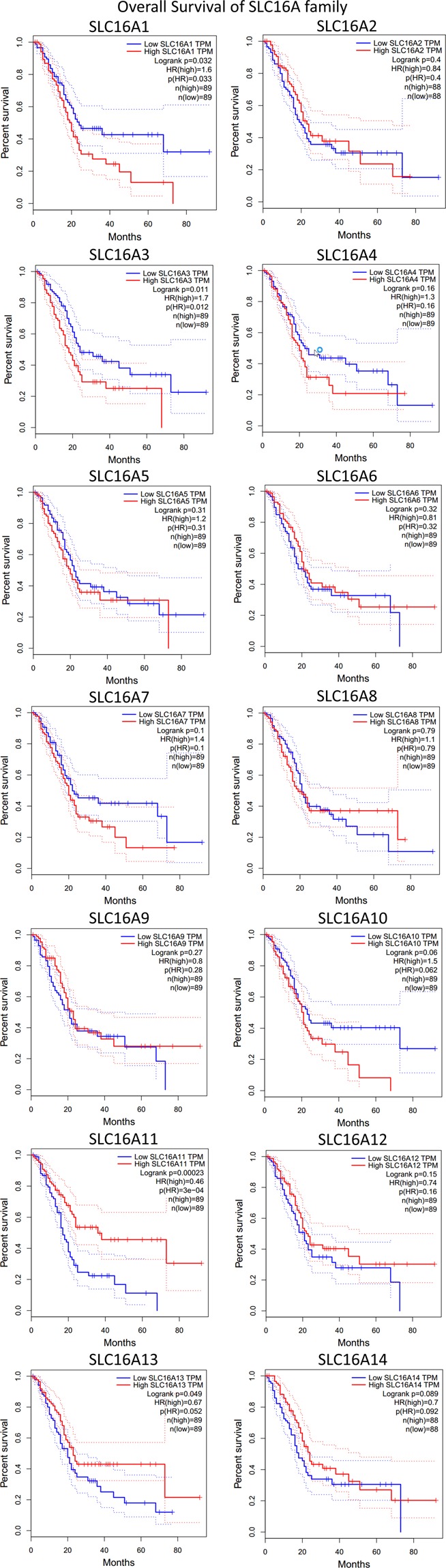
The independent prognostic values of the SLC16A family members in pancreatic cancer for overall survival predictions by Kaplan-Meier analysis. A log-rank p-value < 0.05 was considered statistically significant. The median seq expression level was set as the cutoff for the KM plot. Total patient numbe = 178. The red line indicates high expression, and the blue line indicates low expression.

To date, only a few members of the SLC16A family have been investigated for potential prognostic value in certain cohorts. Thus, we applied Kaplan-Meier analysis with a 95% confidence interval according to RNA-seq data of SLC16A family members from TCGA overall survival data (Fig. 4). A total of 178 pancreatic cancers were divided by the median RNA-seq result (TPM). The median expression level for SLC16A1 could be considered a significant prognosis marker for overall survival time for pancreatic cancer with an HR value = 1.6 and log rank P-value = 0.032. Low expression of SLC16A3 in patients with pancreatic cancer indicated a better survival time (HR = 1.7, log-rank P-value = 0.011). However, SLC16A11 and SLC16A13 are acting as favor prognosis genes for pancreatic cancer if higher than median expression in TCGA cohort. (HR = 0.46, log rank P-value = 0.00023 and HR = 0.67, log rank P-value = 0.049). Other family members in the SLC16A family showed negative results. Because SLC16A11 and SLC16A13 are currently only predicted to be protein-coding, we did not include them in the following bioinformatics analysis. However, in our selected validation cohort (GSE28735), which contains 45 pancreatic cancer patients with overall survival time, we observed that SLC16A1 and SLC16A3 only demonstrate the same trend if separated the patients into high and low groups at median expression. There is no statistical difference for either SLC16A1 (HR = 1.31, P-value = 0.3181) or SLC16A3 (HR = 1.14, P-value = 0.6232) (Figure S4).

### IHC predicted pattern for potential clinical applications

**Figure 5:**
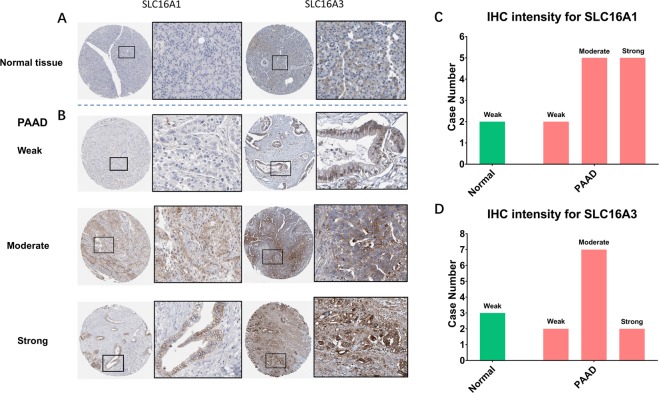
The IHC expression pattern of SLC16A1 and SLC16A3 in normal pancreatic tissue and pancreatic cancer. (**A**) Normal pancreas tissue. **(B**) Representative IHC of PAAD with weak, moderate and strong staining. The black rectangle indicates a higher magnification of the indicated area in the picture. (**C**) Bar chart of IHC staining intensity of SLC16A1 for pancreatic cancer (total of 14 cases). (**D**) Bar chart of the IHC staining intensity of SLC16A3 for pancreatic cancer (total of 14 cases).

AsSLC16A1 and SLC16A3 were positive with our KM overall survival analysis, we further validated the IHC pattern in the HPA to simulate the clinical application by pathology. Normal pancreatic tissue staining of SLC16A1 and SLC16A3 showed negative staining in the nucleus, cytoplasm and cell membrane (Fig. 5A). In pancreatic cancer samples, there were distinguishable patterns for SLC16A1, as only strong staining was positive on the cell membrane. Weak and moderate staining were both negative on the cell membrane. For SLC16A3, the weak pattern was located mainly in the nucleus, but moderate and strong patterns were clearly located on the cell membrane. This unique IHC staining form in pancreatic cancer could help the clinic predict clinical outcome and distinguish cancer from normal tissue (Fig. 5B). The relative staining intensity for SLC16A1 was weak (2 cases) for normal tissue and weak (2 cases), moderate (5 cases) and strong (5 cases) for pancreatic cancer cases (Fig. 5C). For SLC16 A3 staining, all 3 cases were weak for normal tissue and weak (2 cases), moderate (7 cases) and strong (3 cases) for pancreatic cancer samples (Fig. 5D).

### DEG identification from GEO datasets

**Figure 6:**
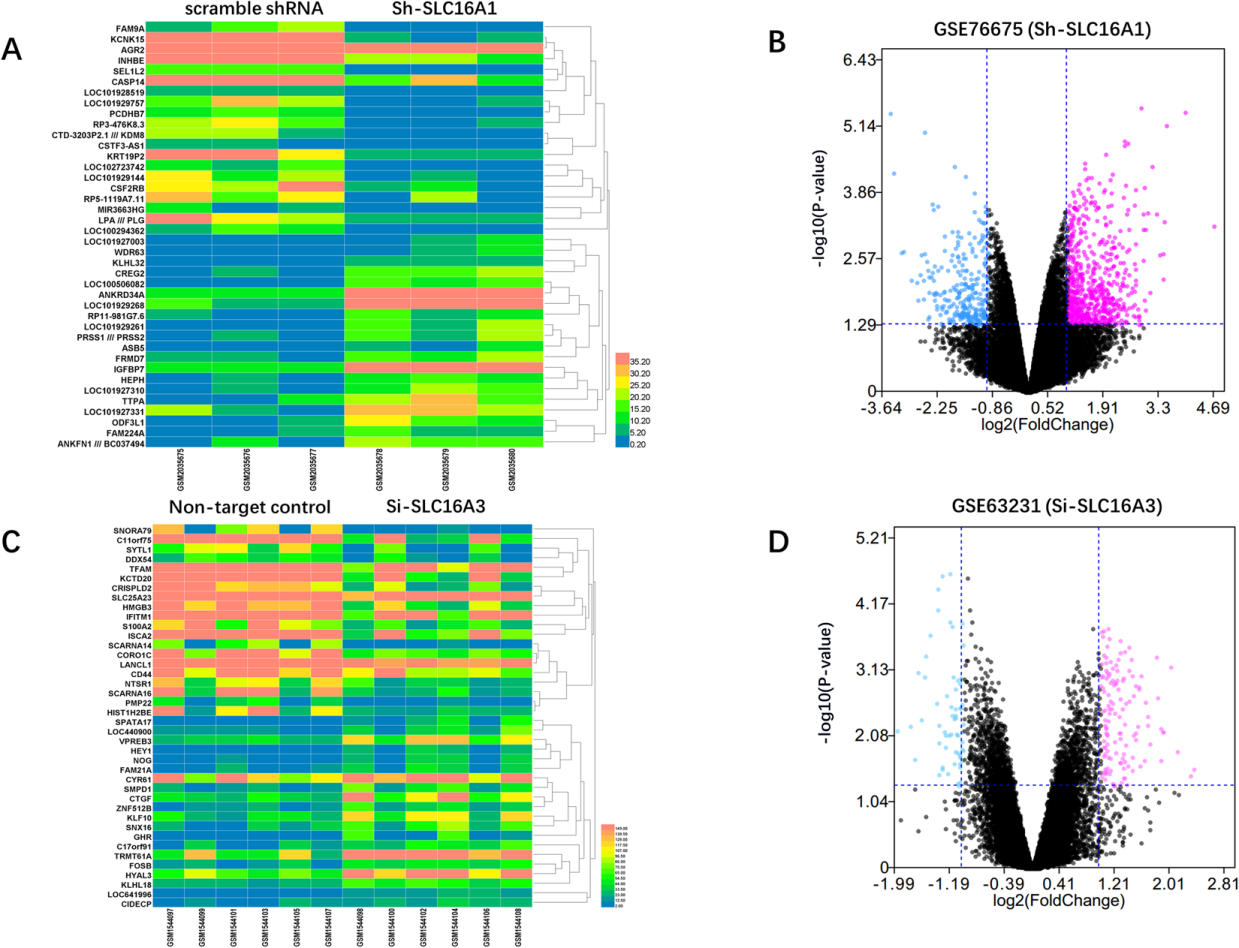
The DEGs of GSE76675 and GSE63231 in the heatmap and volcano plot. (**A**) Heatmap of GSE76675 with the top 20 upregulated and downregulated genes between the scramble shRNA control group and the stable knockdown SLC16A1 group. (**B**) The volcano plot shows the DEG distributions in both up- and downregulated genes. The blue color shows the downregulated genes, while the pink indicates the upregulated genes. (**C**) Heatmap of GSE63231. (**D**) The volcano plot for DEGs in GSE63231.

R software loaded with the limma package was used to identify differentially expressed genes (DEGs) from GSE76675 (SLC16A1) and GSE63231 (SLC16A3) in the indicated subdatasets between the control group and the SLC16A1 and SLC16A3 gene knockdown group. The top 20 upregulated genes and 20 downregulated genes were plotted in a heatmap (Fig. 6A,C). All differentially expressed genes with fold change >1.5 and P-value in both GEO datasets were plotted by a volcano map (Fig. 6B,D). As GSE76675 only contained one cell line with triple replicates, we identified more DEGs than GSE63231 datasets.

### Biological functions and KEGG pathway enrichment analysis

**Figure 7:**
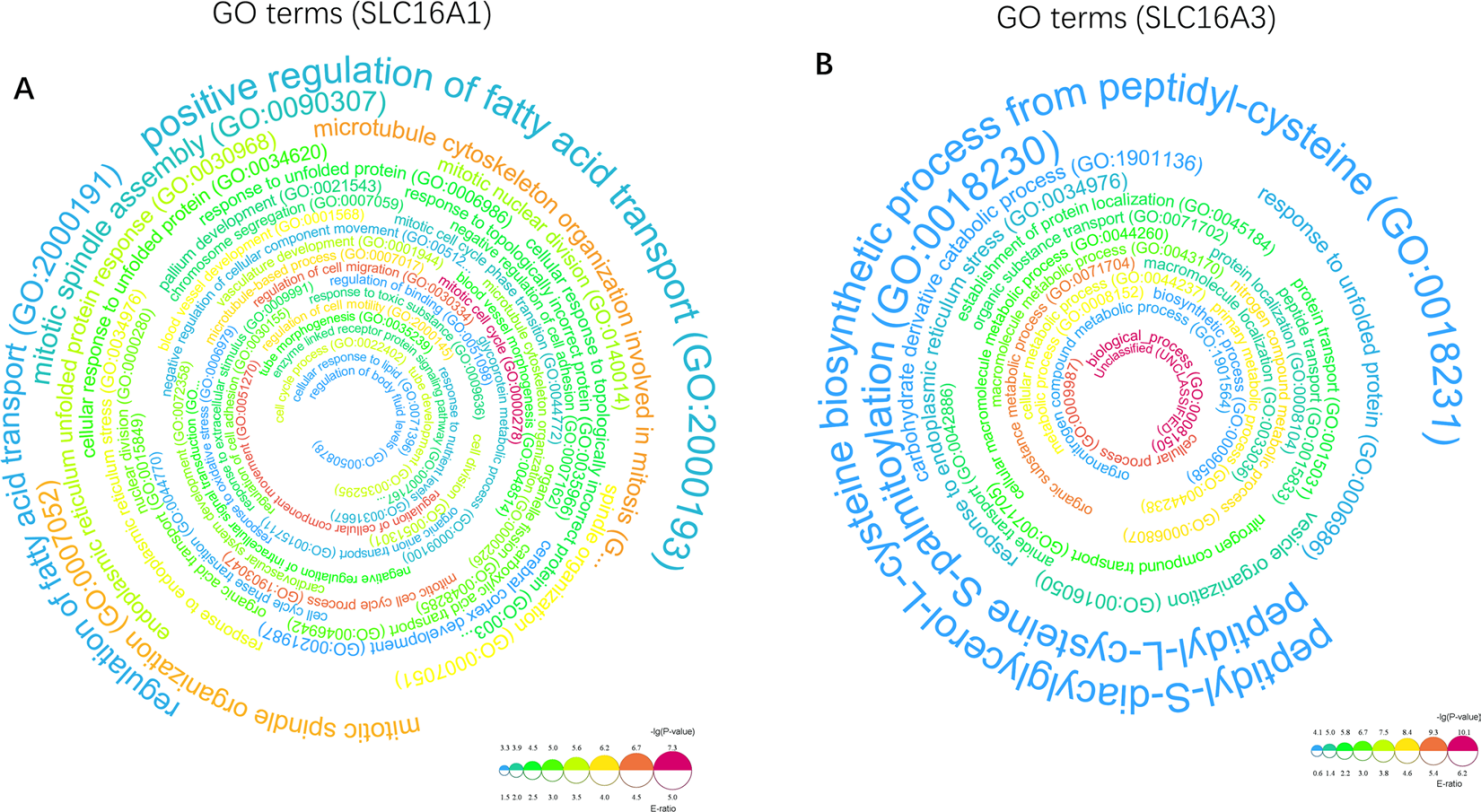
Functional enrichment analysis of Gene Ontology terms. The enrichment ratio P-value for each GO term is calculated and plotted by using Word cloud for the Enrichment Analysis, version 1.025. FDR < 0.05 was set as a cutoff for significance. Larger words indicate a higher enriched ratio, and the word color gradient indicates −lg(p-value). (**A**) All enriched DEGs by GO term analysis for GSE76675. (**B**) All enriched DEGs by GO analysis for GSE63231.

To understand the biological functions behind the DEGs, all DEGs were uploaded into the DAVID database for GO analysis. All enriched GO terms were demonstrated in a circle word map for SLC16A1 (Fig. 7A) and SLC16A3 (Fig. 7B). The top 10 GO terms for SLC16A1 knockdown were positive regulation of fatty acid transport (GO:2000193); regulation of fatty acid transport (GO:2000191); mitotic spindle assembly (GO:0090307); mitotic spindle organization (GO:0007052); microtubule cytoskeleton organization involved in mitosis (GO:1902850); endoplasmic reticulum unfolded protein response (GO:0030968); spindle organization (GO:0007051); mitotic nuclear division (GO:0140014); cellular response to unfolded protein (GO:0034620) and cellular response to topologically incorrect protein (GO:0035967). The top 10 GO terms for SLC16A3 knockdown were peptidyl-S-diacylglycerol-L-cysteine biosynthetic process from peptidyl-cysteine (GO:0018231); peptidyl-L-cysteine S-palmitoylation (GO:0018230); response to unfolded protein (GO:0006986); carbohydrate derivative catabolic process (GO:1901136); response to endoplasmic reticulum stress (GO:0034976); vesicle organization (GO:0016050); protein transport (GO:0015031); establishment of protein localization (GO:0045184); peptide transport (GO:0015833); and nitrogen compound transport (GO:0071705).

**Figure 8:**
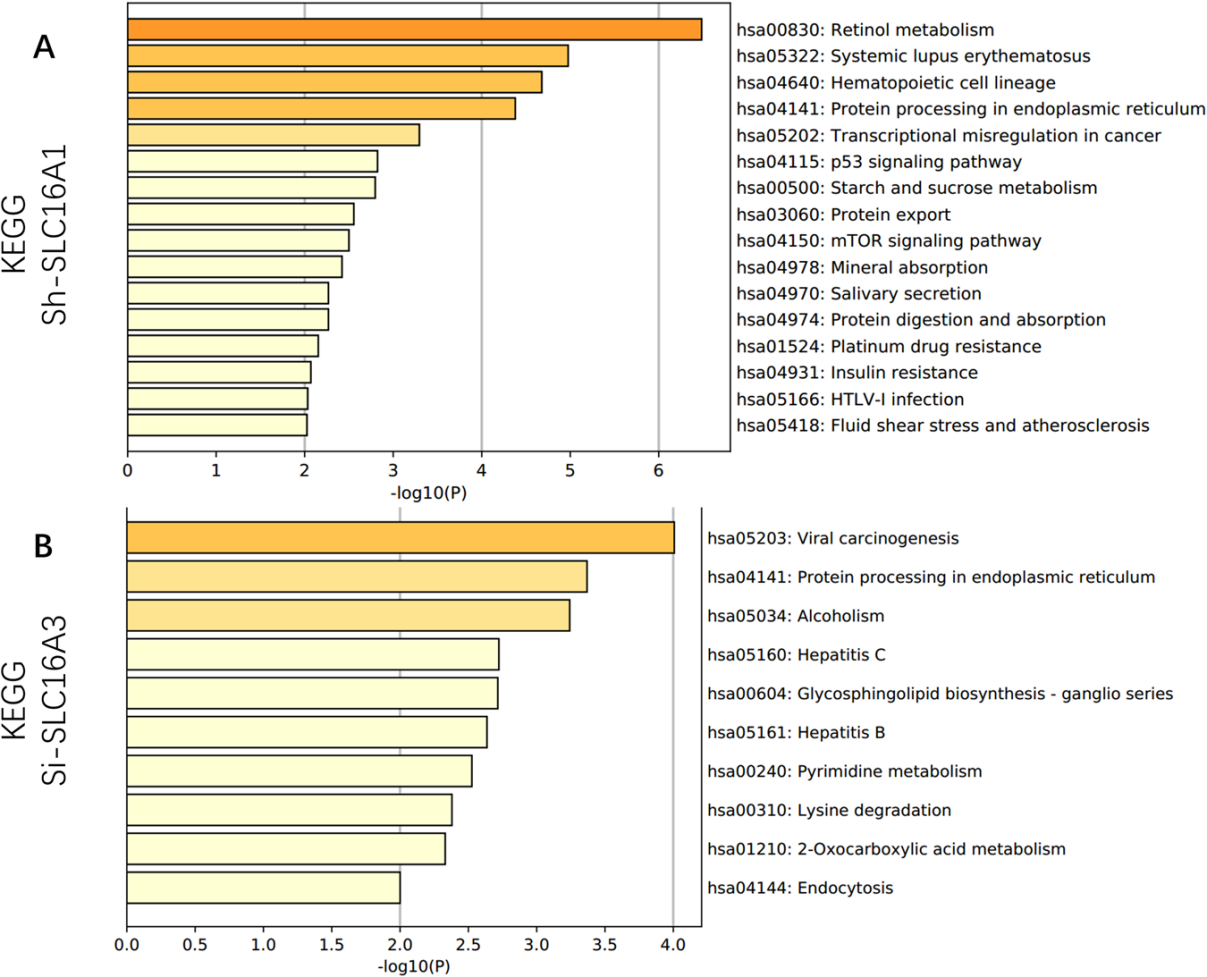
KEGG biological pathway enrichment analysis of DEGs from (**A**) the silencing effect of SLC16A1 in GSE76675 and (**B**) the silencing effect of SLC16A3 in GSE63231. Metascape was used to obtain the KEGG enrichment results and further displayed the KEGG terms on the x-axis that were significantly enriched by −log10 (p-value).

For KEGG pathway enrichment analysis, the regulatory network of KEGG pathways by SLC16A1 knockdown is shown in Fig. 8A. The top 10 enriched KEGG pathways were hsa00830 Retinol metabolism; hsa05322 Systemic lupus erythematosus; hsa04640 Hematopoietic cell lineage; hsa04141 Protein processing in the endoplasmic reticulum; hsa05202 Transcriptional misregulation in cancer; hsa04115 p53 signaling pathway; hsa00500 Starch and sucrose metabolism; hsa03060 Protein export; hsa04150 mTOR signaling pathway; and hsa04978 Mineral absorption.

For SLC16A3, downregulated SLC16A3 mainly participated in the KEGG pathway displayed in Fig. 8B. Only 10 KEGG pathways were identified as follows: hsa05203 Viral carcinogenesis; hsa04141Protein processing in endoplasmic reticulum; hsa05034 Alcoholism; hsa05160 Hepatitis C; hsa00604 Glycosphingolipid biosynthesis - ganglio series; hsa05161 Hepatitis B; hsa00240 Pyrimidine metabolism; hsa00310 Lysine degradation; hsa01210 2-Oxocarboxylic acid metabolism; hsa04144 Endocytosis.

### Understanding the protein-protein interaction (PPI) network of SLC16A1 and SLC16A3

The PPI network helps biologists understand the underlying molecular mechanisms behind the group of regulated genes. We analyzed all DEGs in the PPI network for those two types of proteins using the STRING database. As 1639 nodes and 10,600 edges were identified if we set the minimal interaction score at >0.4, we further used a highly minimal interaction score for the SLC16A1 inhibition study of >0.995 to make the core PPI networks clearer (Figure S1A). The enriched GO biological process terms were as follows: GO:0000278 mitotic cell cycle, FDR = 1.08e-10; GO:0007049 cell cycle, FDR = 2.05e-10; GO:1903047 mitotic cell cycle process, FDR = 3.29e-10; GO:0022402 cell cycle process, FDR = 8.54e-10; GO:0051301 cell division FDR = 1.57e-08; GO:0000280 nuclear division FDR = 2.03e-07; GO:0140014 mitotic nuclear division FDR = 5.81e-07; GO:0048285 organelle fission, FDR = 6.25e-07; GO:0007059 chromosome segregation, FDR = 7.48e-07; and GO:0007017 microtubule-based process, FDR = 1.45e-05. The PPI-based enriched GO terms strongly suggested that SLC16A1 is involved in cancer cell proliferation and the cell cycle.

The PPI network from DEGs of SLC16A3 knockdown research showed 1409 nodes and 5488 edges at a minimal interaction score of >0.4. To clearly demonstrate the result, we use a minimal interaction score of >0.995 (Figure S1B). The enriched PPI biological process GO terms were GO:0071704 organic substance metabolic process, FDR = 0.0448; GO:0044238 primary metabolic process, FDR = 0.0448; GO:0044237 cellular metabolic process, FDR = 0.0448; GO:0008152 metabolic process, FDR = 0.0448; and GO:0006807 nitrogen compound metabolic process, FDR = 0.0448. These findings confirmed that SLC16A3 is a key regulator in the metabolic process of pancreatic cancer.

## Discussion

In recent years, precision treatment has made tremendous improvements in certain kinds of cancer^37^, such as breast cancer and lung cancer, and prognosis biological indicators, such as estrogen receptor (ER) and human epidermal growth factor receptor 2 (HER-2), have been deeply investigated as well^38^. However, the mortality of pancreatic cancer is still slightly increasing due to the lack of effective therapy targets, as the key molecular signatures in pancreatic cancer are still not well characterized^39^.

Because pancreatic cancer is confirmed to be highly dependent on glucose metabolism, the SLC16A family, which encodes monocarboxylate transporter proteins, may be ideal targets for cancer therapy. Therefore, in our study, we systematically evaluated their relevance as therapeutic targets for the diagnosis, prognosis, and cancer treatment of pancreatic adenocarcinoma. Previous research has demonstrated that SLC16A1 is a direct Wnt target and determines tumor sensitivity to anticancer drugs in colon cancer and could also be used as a prognostic marker for NSCLC independently^40,41^. The SLC16A2 mutations were found to be associated with Allan-Herndon-Dudley syndrome^42^. SLC16A3 DNA methylation and MCT4 protein levels were found to be prognostic markers for kidney cancer and thyroid cancer^43,44^. A report in JAMA Oncology demonstrated the association between SLC16A5 and cisplatin-induced ototoxic effects in testicular cancer^45^. A Japanese group collected 210 patients with colorectal cancer and, through a prospective study, showed that the MCT4 expression level was statistically related to VEGF expression and tumor growth and progression^12^. Aberrant expression of the MCT family was found in breast cancer, glioblastoma, prostate cancer, clear cell renal cell carcinoma and adrenocortical carcinoma^43,46–48^. Ever since the function of transporting L-lactate by the MCT family was identified, investigations of MCTs as diagnostic biomarkers or prognostic factors for cancer have emerged. In cervical cancer, MCT1/4 is strongly associated with tumor progression^49^. In addition, the clinical outcome of invasive ductal breast carcinoma could be predicted by MCT1^50^. MCT2 is an ideal biomarker for pathologists to distinguish prostate tumor malignant transformation, as it is not expressed in normal tissue but is highly expressed in neoplastic tissue^47^. There are also reports on MCT4 as a novel prognostic marker in pancreatic cancer, hepatocellular cancer and ovarian cancer^29,51,52^. Moreover, Bovenzi *et al*. carried out a meta-analysis with the conclusion that higher MCT4 indicates worse clinical outcome during a pan-cancer analysis^53^. As there are few studies on other members of the SLC16A family besides only rare evidence that MCT8 and MCT10 may regulate thyroid hormone transportation^54,55^, their roles in cancer are still far from being known and require further investigation^5^.

The results from our study showed that SLC16A1 and SLC16A3 RNA level expression was significantly increased in PAAD samples compared with normal tissues according to the Oncomine multiple pan-cancer analysis, indicating that these two genes widely participate in cancer biological behavior. Additionally, the results from the studies (Logsdon, Pei and Segara) in the Oncomine database using microarrays were the same as the RNA-seq data. Similar results (Badea, Lacobuzio-Donahue, Pei and Segara) for SLC16A3 were observed. SLC16A1, SLC16A2, SLC16A3 and SLC16A4 have relatively higher expression compared to other members of the SLC16A family. Although SLC16A7 was significantly decreased in most investigations, its RNA-Seq expression did not show a difference between the pancreatic cancer samples and normal tissues. However, its role in cancer is still unknown and worthy of deep exploration. For prognosis indicator validation, we found that SLC16A1, SLC16A3 and SLC16A13 have potential via KM analysis. For SLC16A11, this gene is more likely related with human diabetes as single nucleotide polymorphism of SLC16A11 making genomic variants in different populations. In addition, the protein-coding for SLC16A11 is also in doubt. As neither the protein structure nor the functionality of SLC16A13 has been well characterized by the research field, we did not use those genes for informatics molecular function analysis. However, in our validation cohort (GSE28735), we did not find a significant difference but rather the same survival trend for either SLC16A1 or SLC16A3, which may be explained by the low patient number (total of 45 cases) included. We identified the chain biological interactions by two independent investigations through downregulated SLC16A1 and SLC16A3. All gene alteration networks were interpreted using our comprehensive bioinformatics to help us understand the underlying mechanism for the GO terms and KEGG pathways for those two genes. These significantly enriched GO terms and KEGG pathways could help us deeply understand the function of SLC16A1 and SLC16A3, which are involved in the occurrence and progression of pancreatic cancer. Moreover, our research has a limitation in that the bioinformatics analysis for SLC16A1 was based on the RNA-seq data from the knockdown of SLC16A1 in human breast cancer, which weakened the biological process predictions of pancreatic cancer. Although we know that breast cancer is also a highly glycolytic malignant tumor, our bioinformatics prediction of SLC16A1 still requires more experimental studies for validation. Our major findings for SLC16A1 regarding its participation in gene networks showed that SLC16A1 was a key controller of the cell cycle and mitosis, strongly suggesting its oncogene role in promoting cancer cell proliferation. SLC16A3 had a major effect on the metabolic process of pancreatic cancer. For KEGG pathways, SLC16A1 is mainly involved in the P53 signaling pathway, mTOR signaling pathway and platinum drug resistance, which further confirmed its role in cancer. SLC16A3 participated in viral carcinogenesis and protein processing in the endoplasmic reticulum, indicating its important impact on cancer development. Taken together, our findings, including the IHC pattern, demonstrated that SLC16A1 and SLC16A3 could be potentially ideal prognosis makers and therapy targets for patients with pancreatic cancer as treatment strategies. Our results also support previous findings in various cancers, but further in-depth studies including a large cohort of patients should be carried out to validate the molecular pathways and possibility as prognostic markers.

The major challenge in pancreatic cancer is the identification of subgroups of patients who will benefit from targeted therapy; thus, an effective indicator is urgently needed. We aim to understand the clinical value of the SLC16A family in pancreatic cancer and the molecular mechanism from big data. Additionally, we hope that our findings may offer new perspectives in future research and clinical applications for patients with pancreatic cancer. However, more research attention should be paid to the association between SLC16A1, SLC16A3 and pancreatic cancer as new indicators in large patient cohorts.

## Supplementary information

Supplementary figures with legends.

## Data Availability

Research Data are shared with reasonable request.
