# Supplementary material for: Comprehensive analysis of the SLC16A gene family in pancreatic cancer via integrated bioinformatics
Source: Sci Rep. 2020 Apr 30;10:7315. doi: 10.1038/s41598-020-64356-y (PMC7193566; doi:10.1038/s41598-020-64356-y)
Supplement: Supplementary file 1 — Supplementary figures with legends. [file 41598_2020_64356_MOESM1_ESM.pdf]

Comprehensive analysis of the SLC16A gene family in pancreatic cancer via integrated bioinformatics

Running title: The SLC16A family in pancreatic cancer prognosis

Shan Yu[1#*], Yanshuang Wu[2#], Chunlong Li[3], Zhaowei Qu[4], Ge Lou[1], Xiaorong Guo[1], Jingjing Ji[1], Nan Li[5], Mian Guo[6], Maomao Zhang[7], Lei Lei[2.8], Sheng Tai[3*]

1Department of Pathology, the Second Affiliated Hospital of Harbin Medical University, Harbin 150001, China

2Department of Histology and Embryology, Harbin Medical University, Harbin 150001, China

3Department of Hepatobiliary and Pancreatic Surgery, the Second Affiliated Hospital of Harbin Medical University, Harbin 150001, China

4Department of Hepatobiliary and Pancreatic Surgery, Harbin Medical University Cancer Hospital, Harbin 150001, China

5Department of Pathology, the Fourth Affiliated Hospital of Harbin Medical University, Harbin 150001, China

6Department of Neurosurgery, the Second Affiliated Hospital of Harbin Medical University, Harbin 150001, China

7The Key Laboratory of Myocardial Ischemia, Department of Cardiology, the Second Affiliated Hospital of Harbin Medical University, Harbin 150001, China

8The Key Laboratory of Preservation of Human Genetic Resources and Disease Control in China, Harbin Medical University, Ministry of Education, Harbin 150001, China

#Shan Yu and Yanshuang Wu contribute equally to the article

Correspondence to Shan Yu, Department of Pathology, the Second Affiliated Hospital of Harbin Medical University, 246 Xuefu Street, Nangang District, Harbin, 150001, China. Email: yushan@hrbmu.edu.cn and Sheng Tai, Department of General Surgery, the Second Affiliated Hospital of Harbin Medical University, 246 Xuefu Street, Nangang District, Harbin, 150001, China. Email:taisheng1973@163.com

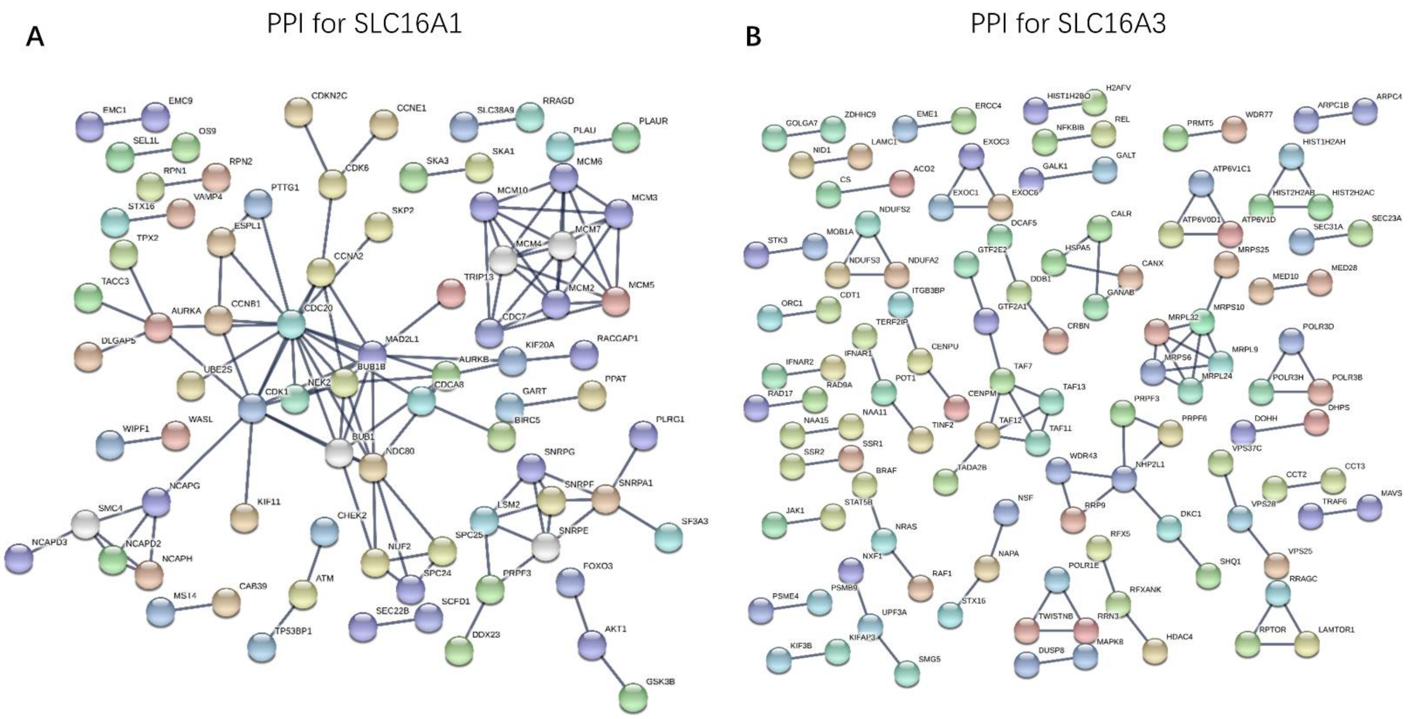

Figure S1: Protein-protein interaction (PPI) network of DEGs. (A) Protein-protein interaction network of DEGs with a connectivity score >0.995 (GSE76675). (B) GSE63231. All disconnected nodes are hidden.

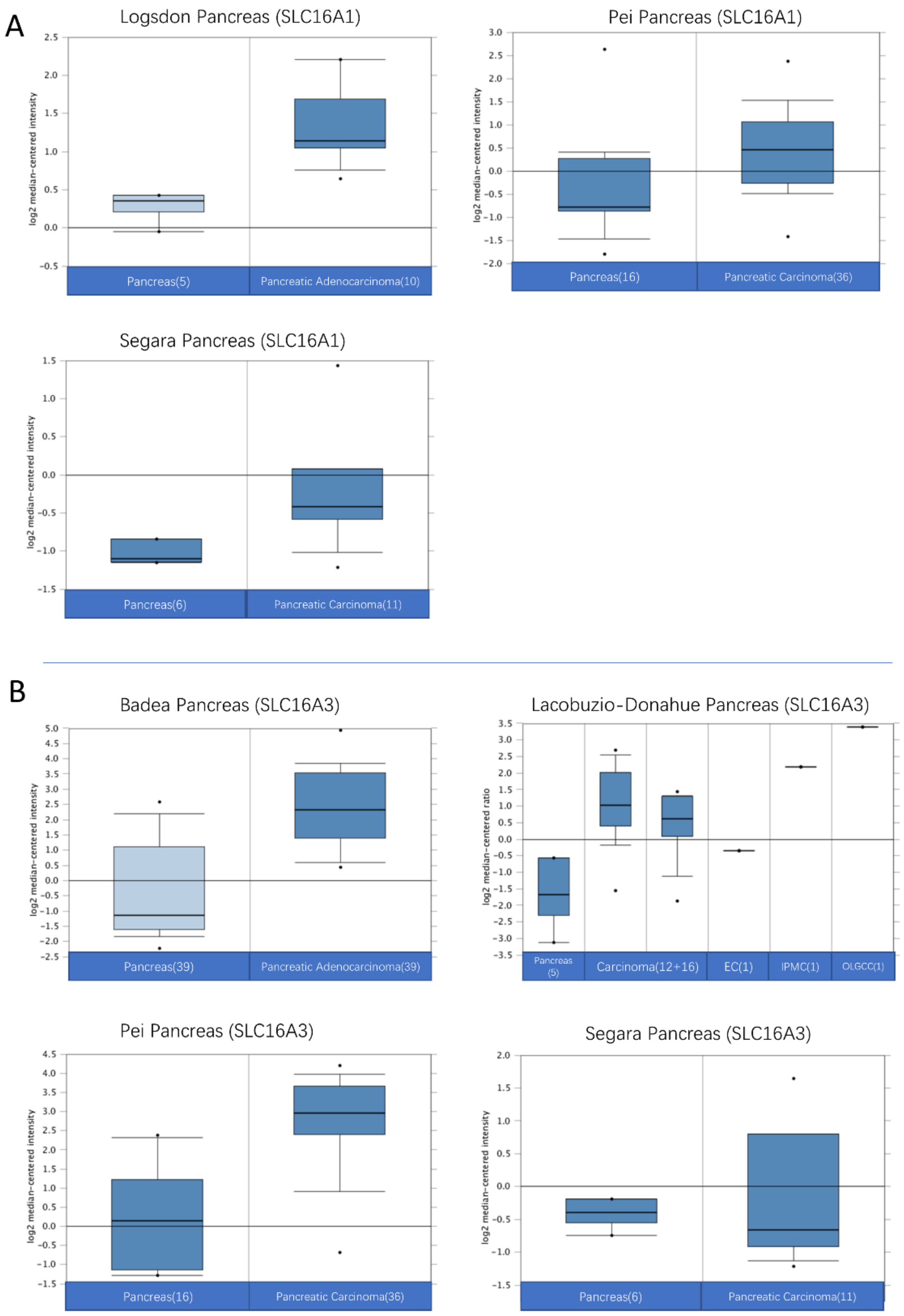

Figure S2: The bar chart of the expression of SLC16A1 and SLC16A3 in pancreatic cancer investigations from the Oncomine database according to our selection criteria. The pancreatic cancer subtypes with P-values (P<0.05) and expression compared vs normal tissues (>1.5-fold) and expressed gene rank in the top 10%. (A) Research on SLC16A1 in the Logsdon, Pei and Segara studies. (B) Research on SLC16A3 in the Badea, Lacobuzio-Donahue (EC=endocrine carcinoma, IPMC=intraductal papillary-mucinous carcinoma, OLGCC=osteoclast-like giant cell carcinoma), Pei and Segara studies. All expression levels of the target genes were determined using the log2 median-centered intensity or ratio.

A

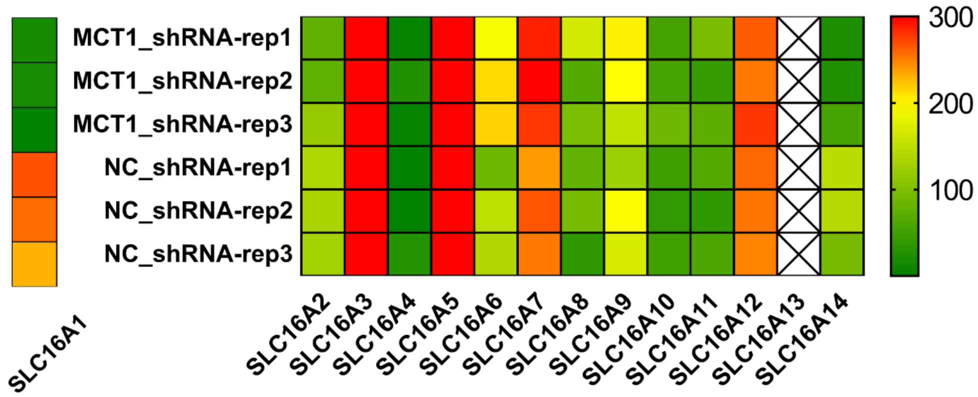

B

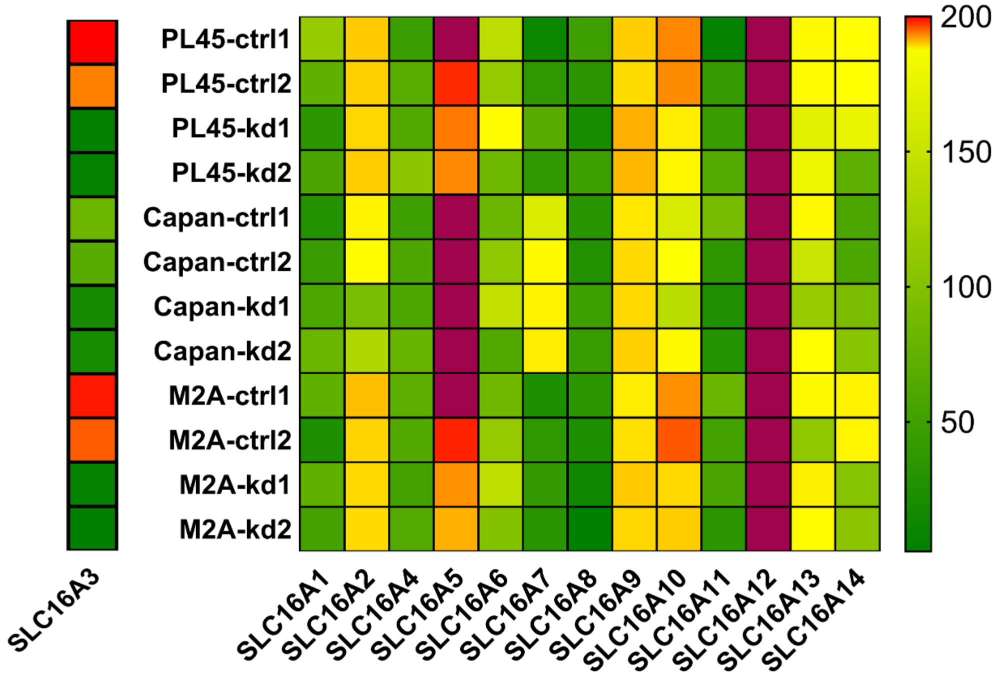

Figure S3: Heatmap of the expression alteration for the SLC16A family with silenced SLC16A1 or SLC16A3. (A) Heatmap of RNA-seq from data provided by the GSE76675 dataset (SUM149, breast cancer cell line). (B) Heatmap of RNA-seq from data provided by the GSE63231 dataset (PL45, Capan, and M2A are three pancreatic cancer cell lines). All heatmaps were plotted with the raw signal intensity. White cells marked with an X indicate no signal detected.

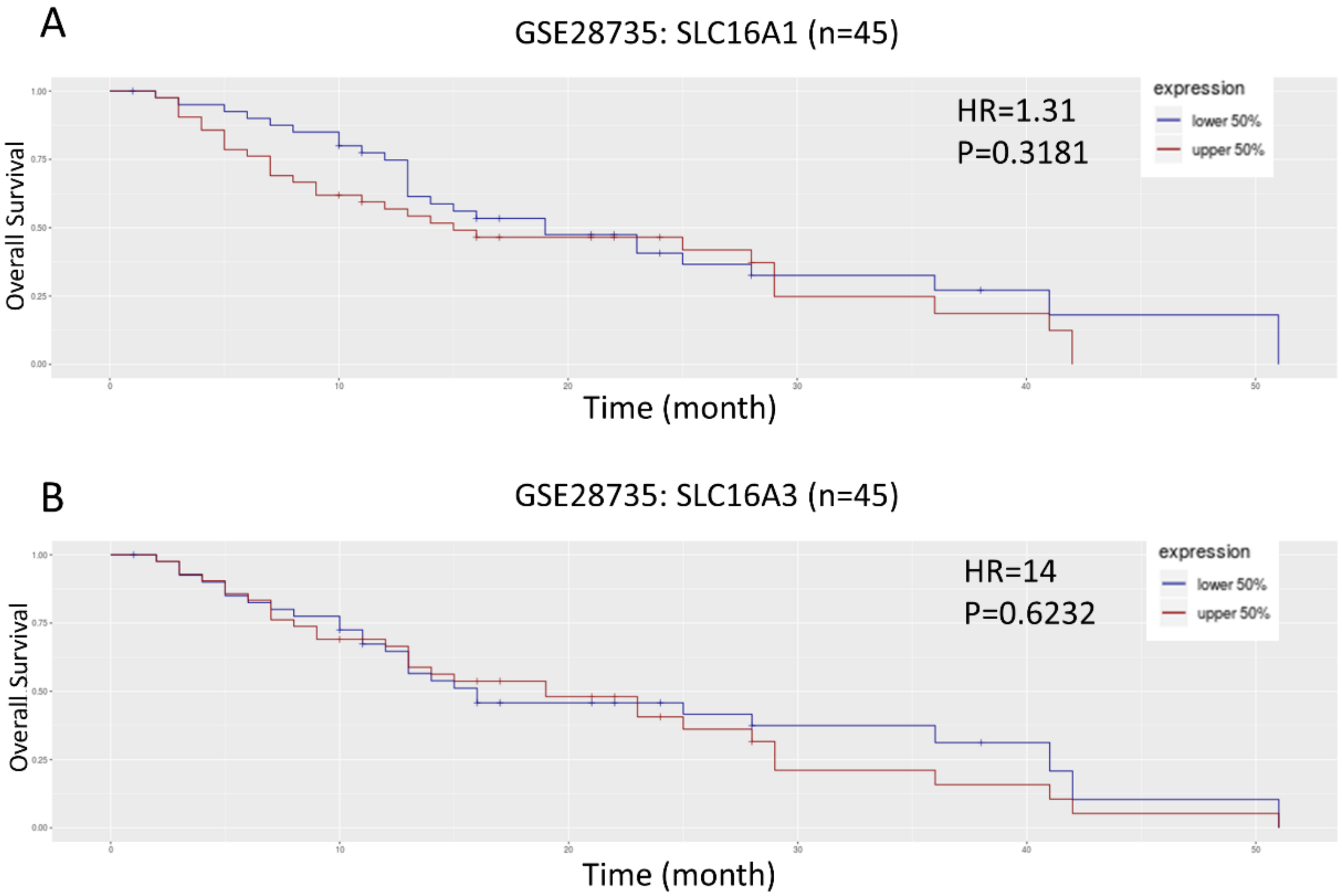

Figure S4: The overall survival KM plot of GSE28735. The median expression of the microarray intensity was adopted as the cutoff for the plot. The blue line indicates less than 50%; the red line indicates greater than 50% of cases. (A) KM plot of SLC16A1. (B) KM plot of SLC16A3.
